# Duodenocaval Fistula: A Spontaneous Complication of Chemoradiation Therapy in Stage III Ovarian Cancer

**DOI:** 10.7759/cureus.25031

**Published:** 2022-05-15

**Authors:** Marc Assaad, Rachelle Hamadi, Khalil El Gharib, Racha Abi Melhem, Yevgeniy Skaradinskiy

**Affiliations:** 1 Internal Medicine, Staten Island University Hospital, New York City, USA; 2 Oncology, Staten Island University Hospital, New York City, USA

**Keywords:** bevacizumab toxicity, chemoradiation therapy, perforated duodenal ulcer, retroperitoneal tumor, ovarian cancer, duodenocaval fistula

## Abstract

Duodenocaval fistula (DCF) is a rare entity which is sparsely described in the literature. Few etiologies have been listed including chemoradiation therapy. Early recognition may reduce the high mortality rate. We describe the case of a 63-year-old woman with a history of stage III ovarian cancer treated with cytoreductive surgery and adjuvant chemotherapy, including bevacizumab, who presented to the hospital because of fresh blood per rectum. One month earlier, the patient was admitted to the intensive care unit because of hemorrhagic shock secondary to a necrotic duodenal ulcer and was treated with cauterization. The patient was stable when discharged home, however, she was readmitted to the hospital because of hematemesis and hematochezia and was again in hemorrhagic shock for which the patient was urgently transfused. An abdominal computerized tomography (CT) angiography demonstrated locules of air within the intrahepatic and infrahepatic inferior vena cava (IVC), as well as evidence of communication with the duodenal lumen, and a thrombus within the IVC. The patient was evaluated by the surgical oncology and vascular teams, who deemed the patient inoperable. Our case describes ovarian malignancy, treated by radiation, leading to duodenitis, with subsequent ulcer formation. The co-administration of bevacizumab delayed gastric healing and promoted ulcer perforation favoring fistula formation.

## Introduction

Duodenocaval fistula (DCF) is a rare entity that is sparsely described in the literature. Few etiologies have been listed, some of which incriminate chemoradiation therapy as a causative factor; prompt recognition and management of this entity are paramount as it harbors high morbidity and mortality rate as high as 40% [1,2]. Herein, we report the case of a 63-year-old female patient who presented with gastrointestinal bleeding, later diagnosed secondary to duodenocaval fistula.

## Case presentation

A 63-year-old woman with stage III ovarian cancer was treated with cytoreductive surgery and six cycles of adjuvant carboplatin/paclitaxel, four of them including bevacizumab that she received every three weeks over the last four months, and was presented to the hospital because of fresh blood per rectum. One month before this presentation, the patient was admitted to the intensive care unit for hemorrhagic shock secondary to a 3 cm necrotic duodenal ulcer treated with cauterization. Her hospital course was complicated by septic shock secondary to candidemia and *Escherichia coli* bacteremia treated with caspofungin and piperacillin/tazobactam. The patient was stable when discharged home and was prescribed proton pump inhibitors twice daily; however, after 10 days, she was readmitted to the hospital for hematemesis and again for hematochezia. She was hypotensive with a systolic blood pressure of 90 mmHg, tachycardic to 150 beats per minute; findings were consistent with another hemorrhagic shock. Initial investigations revealed a hemoglobin level of 3.6 g/dL for which, the patient received a total of six units of packed red blood cells.

**Figure 1:**
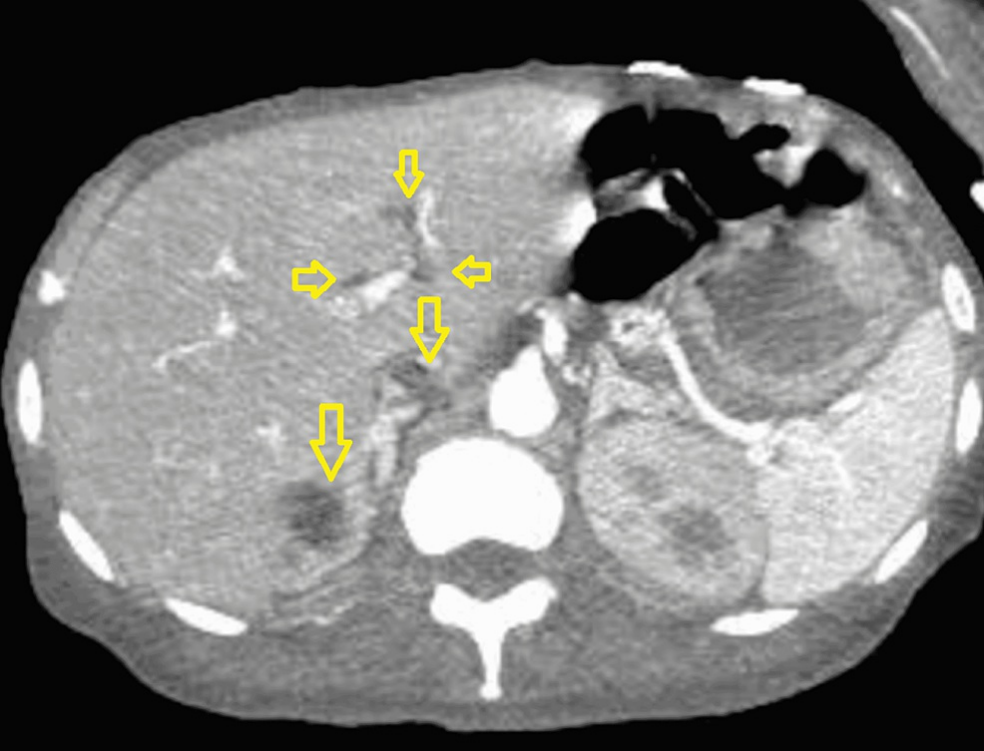
Computed tomography scan of the abdomen showing gas (hypodensities) in the hepatic portal system (arrows) secondary to duodenocaval communication.

**Figure 2:**
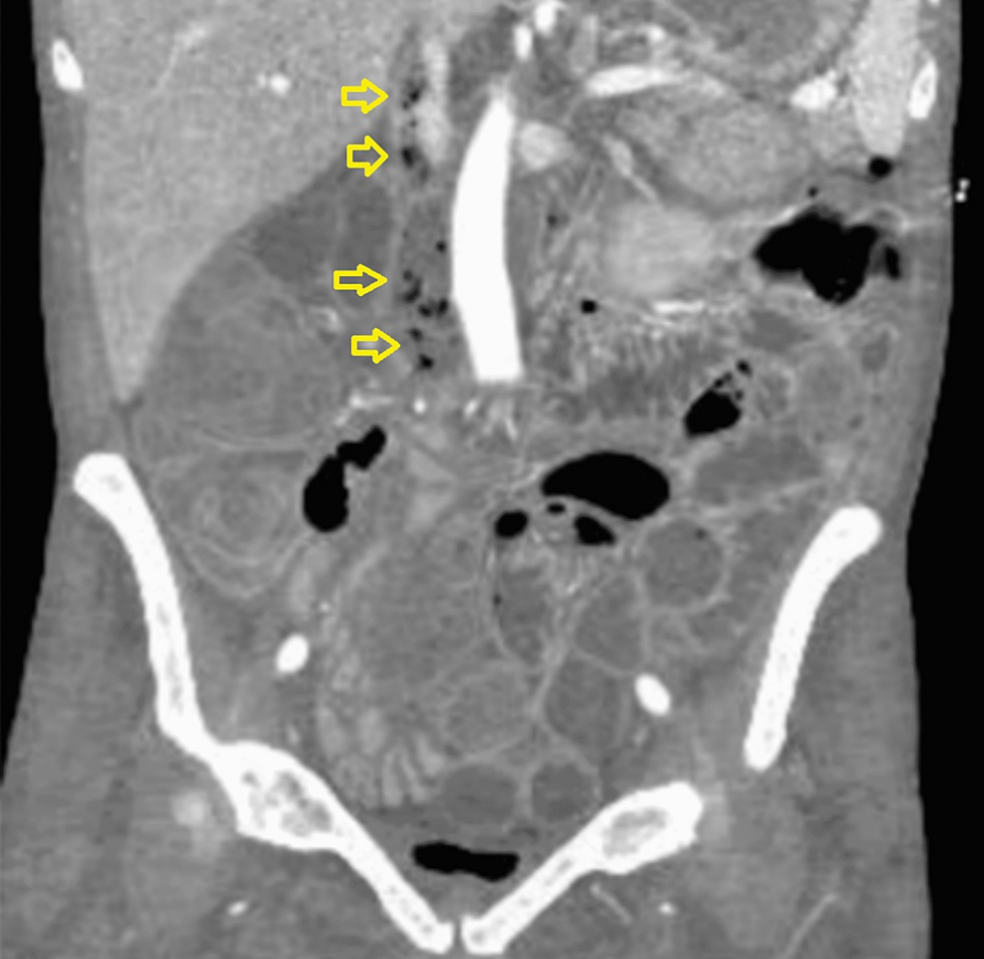
Computed tomography scan of the abdomen and pelvis view showing hypodensities and locules of air (arrows) within the intrahepatic and infra hepatic inferior vena cava.

An abdominal computerized tomography (CT) angiography demonstrated hypodensities and locules of air within the intrahepatic and infrahepatic inferior vena cava (IVC), as well as evidence of communication with the duodenal lumen (Figures 1, 2). The patient was evaluated by the surgical oncology and vascular teams, who deemed the patient inoperable due to her low functional status and extreme debility. The patient was a poor candidate for endovascular approach by interventional radiology due to high mortality and limited life expectancy. The patient was then referred to hospice care for end-of-life measures and passed away within the following week.

## Discussion

A DCF can be created by a foreign body such as toothpick, fishbone, or penetrating abdominal injury [3-6]. Other cases have reported that it is secondary to peptic ulcer disease eroding the duodenal wall and building communication with the adjacent IVC [1,7]. Also, atraumatic DCF has been described following chemoradiation or radiotherapy alone in retroperitoneal tumors. Bevacizumab, an antiangiogenic agent, has also been implicated as it promotes mucosal ulceration and delays its healing, facilitating fistula formation in the gastrointestinal tract [8,9]. Clinical presentation varies from simple abdominal pain, vomiting, and diarrhea to more severe manifestations such as fever, sepsis, and most commonly gastrointestinal bleed [4,7]. This entity can be complicated by bacteremia and fungemia [2,3,6,8], vena cava thrombus [1,5,6], and rarely pulmonary embolism [6]; the most dreaded complication is hemorrhagic shock [1], as noted in our case, and two similar ones illustrated by Perera et al. [10].

Our case describes ovarian malignancy, treated by radiation, having led to duodenitis, with subsequent ulcer formation. The side effects of radiation, including mucositis and ulcerations, can be directly proportional to the dose of radiation given [11]. The coadministration of bevacizumab, an antiangiogenic molecule, delayed gastric healing. The initial treatment of the bleeding ulcer with cauterization was also in favor of more mucosal thinning and necrosis, promoting ulcer perforation and favoring fistula formation. The gold standard diagnostic test remains CT angiography of the abdomen (cavography/phlebography) [2,10]. Most commonly, DCF is treated with laparotomy with or without vagotomy, and less likely with an endoscopic approach. Recently, there has been an increase in the use of endovascular techniques to repair fistulae between vascular and enteric structures [12].

## Conclusions

Duodenal ulceration and fistula formation likely arose from fibrosis and mucosal damage. Discontinuation of radiation or any drug that might potentiate or worsen mucosal ulceration, at least until endoscopic healing is proven, might be beneficial in similar cases for fewer complications to happen.
